# Prepulse inhibition of change-related P50m no correlation with P50m gating

**DOI:** 10.1186/2193-1801-2-588

**Published:** 2013-11-01

**Authors:** Koji Inui, Aki Tsuruhara, Kei Nakagawa, Makoto Nishihara, Minori Kodaira, Eishi Motomura, Ryusuke Kakigi

**Affiliations:** Department of Integrative Physiology, National Institute for Physiological Sciences, Okazaki, 444-8585 Japan; Multidisciplinary Pain Center, Aichi Medical University, Aichi, 480-1195 Japan; Department of Psychiatry, Mie University Graduate School of Medicine, Tsu, 514-8507 Japan

**Keywords:** Auditory evoked magnetic fields, Prepulse inhibition, Sensory gating

## Abstract

Both prepulse inhibition (PPI) of the startle response and P50 sensory gating are important tools to investigate the inhibitory mechanisms of sensory processing. However, previous studies found no or a weak association between these two measures, which may have been due to the different indexes used. We examined the relationship between P50 sensory gating and P50 PPI. P50m sensory gating and PPI of Change-related P50m were assessed in 14 subjects using magnetoencephalography. Concerning P50m sensory gating, the amplitudes of the response to the second click relative to that to the first one were reduced by 43 and 47% for the left and right hemisphere, respectively. Change-related P50m was evoked by an abrupt sound pressure increase by 10 dB in a continuous click train of 70 dB. When this test stimulus was preceded by a click (prepulse) with a weaker sound pressure increase (5 dB) at a prepulse-test interval of 30, 60, or 90 ms, Change-P50m was suppressed by 33 ~ 65% while the prepulse itself elicited no or very weak P50m responses. Although the amplitude of the P50m response to the first click and the amplitude of the Change-P50m test alone response were positively correlated (r = 0.6), the degree of the inhibition of the two measures was not (r = -0.06 ~ 0.14). The neural origin was estimated to be located in the supratemporal plane around the superior temporal gyrus or Heschl’s gyrus and did not differ between P50m and Change-P50m. The present results suggest that P50m and Change-P50m are generated by a similar group of neurons in the auditory cortex, while the mechanisms of P50m sensory gating and Change-P50m PPI are different.

## Background

Sensory gating is an important brain function in which sensory information is screened to allow an individual to focus on the most salient aspects of the sensory environment (Braff et al. 2001; Swerdlow et al. 2001). Understanding this inhibitory mechanism is important since it has been well established that patients with schizophrenia show inhibitory deficits (Swerdlow et al. 2008; Braff 2010), which is considered to be related to the development of their positive symptoms. Sensory gating is measured by prepulse inhibition (PPI) of the startle response or suppression of the auditory evoked P50 component (P50 sensory gating). PPI is a phenomenon in which a weak leading stimulus, or prepulse, inhibits the startling reflexes evoked by a subsequent intense abrupt stimulus (Graham 1975). A blink reflex following an intense sound is commonly used as an index of the startle response. Therefore, it has been referred to as sensorimotor gating. P50 sensory gating is a phenomenon in which an auditory evoked potential component peaking at 50 ms (P50) is suppressed when the same auditory stimulus is repeated. The amplitude of P50 to the first click and that to the second click presented 500 ms after the first one are commonly compared to assess the degree of the inhibition. Although P50 sensory gating is evaluated using electroencephalography, some previous studies used magnetoencephalography (MEG) (Clementz et al. 1997,2003; Edgar et al. 2003; Huang et al. 2003; Thoma et al. 2003; Hanlon et al. 2005; Lu et al. 2007; Hirano et al. 2010). Although both measures are considered to reflect an inhibitory process, previous studies found no or only a weak association between PPI and P50 sensory gating in healthy subjects (Schwarzkopf et al. 1993; Oranje et al. 1999; Light and Braff 2001; Brenner et al. 2004) as well as patients with schizophrenia (Braff et al. 2007; Hong et al. 2007). Since the two measures use different responses as an index, one cortical response and another motor response, the lack of a meaningful correlation in these studies may have been due to methodological issues.

We recently reported that auditory change-related cortical responses were inhibited by a prepulse in a similar manner to the PPI of startle reflexes (Inui et al. 2012). Change-related cortical responses represent sensory-evoked cortical activation specific to the change of a stimulus, and can be very clearly recorded with electroencephalography or MEG. Similar to P50 and startle blink reflexes, the change-related cortical response is preattentive and is elicited without any tasks and without the subject’s attention by any sensory changes including the onset and offset of a stimulus (Yamashiro et al. 2009; Inui et al. 2010a, 2010b; Akiyama et al. 2011; Yamashiro et al. 2011; Ohoyama et al. 2012). Because of similar physiological significance and experimental behavior, the PPI of startle and PPI of change-related cortical responses are considered to share underlying mechanisms (Inui et al. 2012). In addition to the change-related auditory response peaking at 100 ~ 130 ms (Change-N1), we recently demonstrated that an earlier component at approximately 60 ms (Change-P50m) was elicited by an abrupt auditory feature change (Nakagawa et al. 2013). If similar mechanisms exist between PPI and P50 sensory gating, we may identify significant correlations between P50 sensory gating and the PPI of Change-P50 using a similar response, the P50 auditory component.

## Methods

The study was approved in advance by the Ethics Committee of the National Institute for Physiological Sciences, Okazaki, Japan, and written consent was obtained from all subjects. The experiment was performed on 14 (four females and ten males) healthy volunteers, aged 28 – 54 (39 ± 7) years. They were asked to refrain from alcohol, caffeine, and smoking for at least 12 hours prior to the experiment. There were two smokers. All subjects had no history of mental or neurological disorders or substance abuse in the last two years. They were free of medication at testing. They had a hearing threshold lower than 30 dB at 1000 Hz as assessed by an audiometer (AA-71, Rion, Tokyo, Japan).

### Auditory stimuli

**Figure 1:**
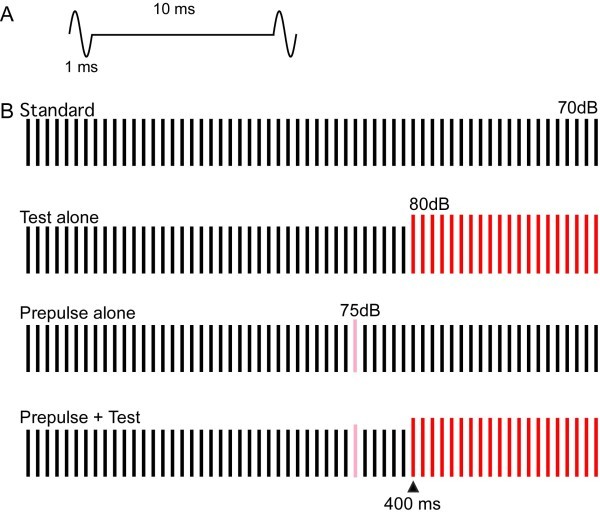
**Auditory stimuli for prepulse inhibition. A)**, a single sine wave 1 ms in duration was repeatedly presented at 100 Hz. **B)**, the standard or background stimulus was a train of click sounds 70 dB SPL in sound pressure and 600 ms in duration. The test stimulus to evoke change-related cortical responses consisted of a similar train of clicks of 70 dB for 400 ms followed by 80 dB clicks for 200 ms. One click of 75 dB was inserted before the test stimulus as a prepulse.

A single sine wave 1 ms in duration was used as a click sound for both P50m sensory gating and PPI experiments. We used a train of the click sounds at 100 Hz (Nakagawa et al. 2013) for the PPI experiment. Four types of sound stimuli were used (Figure 1): 60 repeats of the same click 600 ms in total duration and 70 dB SPL in sound pressure (Standard), 40 standard clicks (400 ms) followed by 20 clicks of 80 dB (Test alone), the Test preceded by one click of 75 dB (Prepulse + Test), and the Standard with Prepulse (Prepulse alone). Sound stimuli were presented binaurally through ear pieces (E-A-Rtone 3A, Aero Company, Indianapolis, IN).

### MEG recordings

Magnetic signals were recorded using a 306-channel whole-head type MEG system (Vector-view, ELEKTA Neuromag, Helsinki, Finland), which comprised 102 identical triple sensor elements. Each sensor element consisted of two orthogonal planar gradiometers and one magnetometer coupled to a multi-superconducting quantum interference device (SQUID) and, thus, provided 3 independent measurements of the magnetic fields. In this study, we analyzed MEG signals recorded from 204 planar-type gradiometers. These planar gradiometers are powerful enough to detect the largest signal just over local cerebral sources. The signals were recorded with a bandpass filter of 0.1-200 Hz and digitized at 1004 Hz. Analysis was conducted from 100 ms before to 300 ms after the onset of the click (P50m sensory gating) or the abrupt increase in sound pressure (PPI). The 100 ms pre-stimulus (or pre-change) period was used as the baseline. Epochs with MEG signals larger than 2.7 pT / cm were rejected from the averaging.

### Procedures

Experiments were conducted in a quiet, magnetically shielded room. Subjects sat in a chair and watched a silent movie on a screen 1.5 m in front of them throughout the experiments. Experiments for PPI and P50m sensory gating were carried out successively in this order with a few minutes rest period for all subjects.

## PPI of change-related P50m

The Prepulse was presented either 30, 60, or 90 ms before the abrupt increase in sound pressure at 400 ms in the click train. Therefore, there were eight stimuli: 1) Standard alone, 2) Test alone, 3) ~ 5) Prepulse alone, and 6) ~ 8) Prepulse + Test. Eight stimuli were presented randomly at an even probability at a trial-trial interval of 800 ms. A total of 150 ~ 155 artifact-free epochs were averaged for each stimulus.

**Figure 2:**
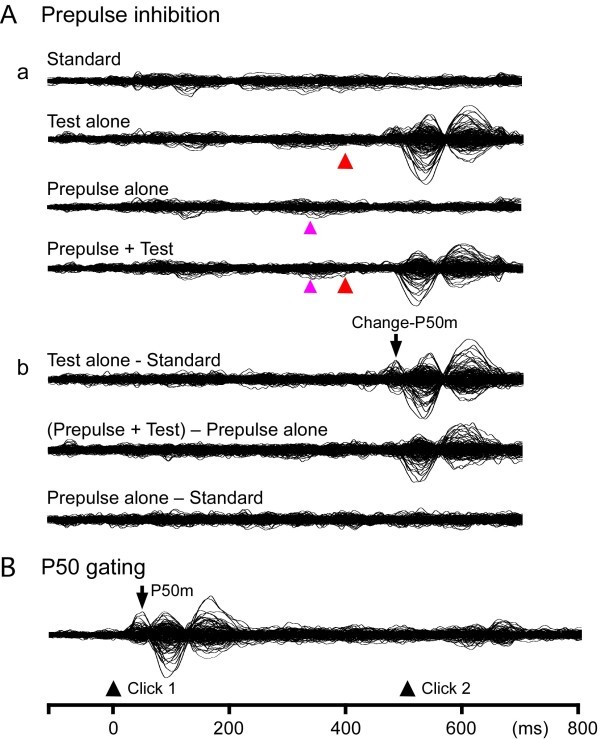
**Magnetic responses to the stimuli.** Superimposed waveforms of all 204 sensors recorded from one representative subject. **A**a, responses to the Standard, Test alone, Prepulse (30 ms) + Test, and Prepulse alone stimuli. **A**b, subtracted waveforms. Red and pink arrowheads indicate the onset of the Test and Prepulse, respectively. **B)**, magnetic responses under the P50m sensory gating paradigm.

Recorded MEG waveforms were subjected to band-pass filtering of 2 ~ 75 Hz and analyzed as previously reported (Inui et al. 2012). In brief, the Test alone response was obtained by subtracting the waveform for the Standard from that for the Test alone stimulus (Figure 2B). Similarly, the Prepulse + Test response was obtained by subtracting the waveform for the Prepulse alone stimulus from that for the Prepulse + Test stimulus. Using the difference waveforms for the Test-alone stimulus, an equivalent current dipole for the magnetic component at around 60 ms, Change-P50m, was estimated for each hemisphere using BESA (NeuroScan, Mclean, VA). The obtained two-dipole model was applied to all subtracted waveforms, and the source strength waveform was used to measure the peak latency and amplitude of Change-P50m. The amplitude of the response was measured from the baseline. The percentage inhibition of the Change-P50m amplitude by the Prepulse (%PPI) was defined as (Test alone response – Prepulse + Test response) / Test alone response * 100.

## P50 sensory gating

A paired stimulation paradigm was used to assess P50m sensory gating. A click 1 ms in duration and 80 dB in sound pressure was used. The click-click interval was 500 ms. The trial-trial interval was 8 ~ 12 s. Epochs of 150 click pairs were averaged. Similar to the above procedures, a two-dipole model was obtained using the waveform evoked by the first click (Click 1). Source strength waveforms were obtained by applying the model to both the waveform for Click 1 and Click 2, and the peak latency and amplitude were measured. The amplitude was measured from the baseline. Percentage inhibition of P50m (P50m%inhibition) was defined as (Click 1 response – Click 2 response) / Click 1 response * 100.

### Statistical analysis

The Change-P50m amplitude and latency were compared among four test responses, Test alone and three Prepulse + Test responses, using a two-way repeated measures ANOVA with Prepulse and Hemisphere as the independent variables. The amplitude and latency of P50m were compared between Click 1 and Click 2 by a two-way ANOVA (Click x Hemisphere). The degree of PPI was compared among three prepulses and between hemispheres by a two-way repeated measures ANOVA. Post hoc comparisons were conducted using Fisher’s least significant difference. The degree of P50m sensory gating was compared between hemispheres by a paired t-test. The location of estimated dipoles was expressed in Talairach coordinates using BESA and Brain Voyager (QX 1.4, Maastricht, the Netherlands). The difference in the source location between the two measures was assessed by a paired t-test for each axis. The correlation coefficient of the inhibition between the two measures was evaluated using Fisher’s Z transformation. When the sphericity assumption was violated, the Greenhouse-Geisser correction coefficient epsilon was used for correcting the degrees of freedom and the F-value and significance probability were then re-calculated. All statistical analyses were performed at the 0.05 level of significance. IBM SPSS Statistics (version 21) was used for statistical analysis. Data are expressed as the mean ± standard deviation (SD).

## Results

### PPI

**Figure 3:**
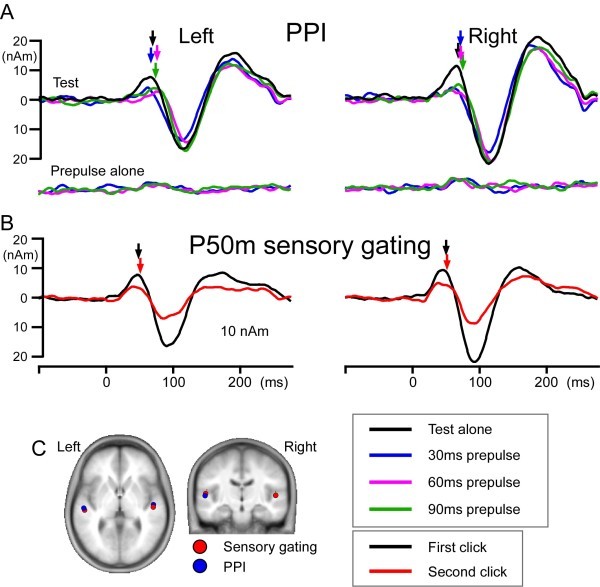
**Grand-averaged source strength waveforms.** Grand-averaged waveforms of Change-P50m **(A)** and P50m **(B)**. The mean peak latencies are indicated by arrows. Upward and downward deflections indicate the source strength of the current directing anterosuperior and posteroinferior, respectively. **C)**, the mean location of the estimated dipole for Change-P50m and P50m superimposed on standard MR images.

The test stimulus, an abrupt increase in sound pressure by 10 dB, evoked a clear response at approximately 60 ms (Change-P50m) in all the 14 subjects tested. Figure 2A shows an example of the recorded (Aa) and subtracted (Ab) waveforms. The dipole for Change-P50m was estimated to be located in the supratemporal plane around the lateral part of Heschl’s gyrus or the superior temporal gyrus (Figure 3C). Figure 3B shows grand-averaged source strength waveforms of the estimated dipole for each stimulus. The mean peak latency and amplitude for each condition are listed in Table 1. The Prepulse alone response could be reliably identified in only one subject, and no further analyses were performed. The results of the two-way ANOVA (Prepulse x Hemisphere) revealed that the amplitude of Change-P50m was significantly different among the four Test responses (F_3,39_ = 21.11, p < 0.001). The Test alone amplitude was significantly greater than the Prepulse + Test amplitude for each Prepulse (lsd = 1.75, df = 39, p < 0.001), which confirmed the inhibitory effects of the prepulse on Change-P50m. Although the Hemisphere was not a significant factor to determine the Change-P50m amplitude (F_1,13_ = 0.253, p = 0.623), a significant Prepulse x Hemisphere interaction was found (F_3, 39_ = 6.1, p = 0.002). The results of each pair comparisons showed that the amplitude of the Test alone response was significantly greater for the right hemisphere than for the left (lsd = 1.69, df = 13, p = 0.02). The peak latency did not differ between hemispheres (F_1,13_ = 0.20, p = 0.66) or among four test responses (F _3,39_ = 1.98, p = 0.132).

**Table 1:** **Peak latency and amplitude of Change-P50m and P50m**

	Latency (ms)		Amplitude (nAm)		Inhibition (%)	
PPI	Lt	Rt	Lt	Rt	Lt	Rt
Test alone	69 ± 10	68 ± 6	9.8 ± 4	12.7 ± 4.4		
30 ms	67 ± 9	70 ± 12	6.1 ± 4.1	4.4 ± 2.9	40 ± 27	65 ± 26
60 ms	74 ± 13	71 ± 11	5.1 ± 3.9	5.7 ± 3.7	44 ± 34	56 ± 20
90 ms	73 ± 9	72 ± 7	6.6 ± 4.2	6.3 ± 3.4	33 ± 35	49 ± 23
**P50 Sensory gating**						
Click 1	52 ± 10	49 ± 10	13.5 ± 6.2	15.2 ± 5.0		
Click 2	53 ± 13	52 ± 13	6.8 ± 3.1	8.5 ± 4.3	43 ± 24	47 ± 23

Values are the mean ± SD.

The results of the two-way ANOVA showed that the degree of the inhibition was significantly stronger for the right hemisphere (F_1,13_ = 12.49, p = 0.004), but was not different among the three Prepulses (F_2,26_ = 1.08, p = 0.355). On average,%PPI was greatest for the right response with the 30-ms Prepulse (65 ± 26%) and was weakest for the left 90-ms Prepulse (33 ± 35%).

### P50 sensory gating

Figure 2B shows an example of magnetic responses to the paired stimulus. The neural source responsible for the P50m component was estimated to be located in the anterolateral part of Heschl’s gyrus or the superior temporal gyrus similar to Change-P50m. Since MEG is not sensitive to sources in deep brain areas or with an intracellular current radial to the brain surface, the present finding does not rule out the involvement of other sources (see Huotilainen et al. 1998). The grand-averaged source strength waveform is shown in Figure 3B. The mean location of the dipole is shown in Figure 3C. The peak latency and amplitude of P50m are listed in Table 1. The results of the two-way ANOVA (Click x Hemisphere) showed that the P50m amplitude was significantly different between Click 1 and Click 2 (F_1,13_ = 46.93, p < 0.001). The P50m amplitude was also slightly different between the hemispheres (F_1,13_ = 3.18, p = 0.098). The amplitude for the right hemisphere was greater for both Click 1 and Click 2 on average. No significant difference was observed in the P50m%inhibition (t_13_ = 0.48, p = 0.636) between the right (43 ± 24%) and left (47 ± 23%) hemispheres. The peak latency did not differ between hemispheres (F_1,13_ = 0.64, p = 0.439) but was slightly shorter for Click 1 (F_1,13_ = 4.51, p = 0.053). The overall P50m latency was 50.6 ± 2.5 ms for Click 1 and 52.9 ± 3 ms for Click 2.

### Relationship between P50m sensory gating and PPI

**Figure 4:**
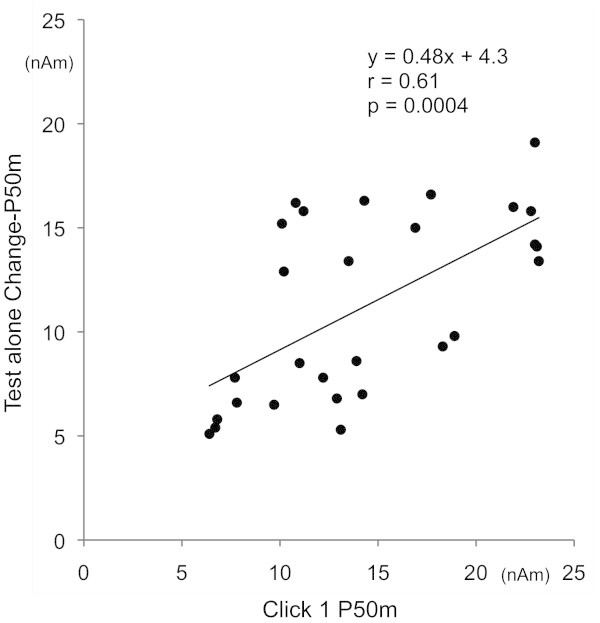
**Correlation of the baseline amplitude between Change-P50m and P50m.** Plots show the relationship between the amplitude of P50m for the first click (x axis) and that of Change-P50m for the Test alone response (y axis).

**Figure 5:**
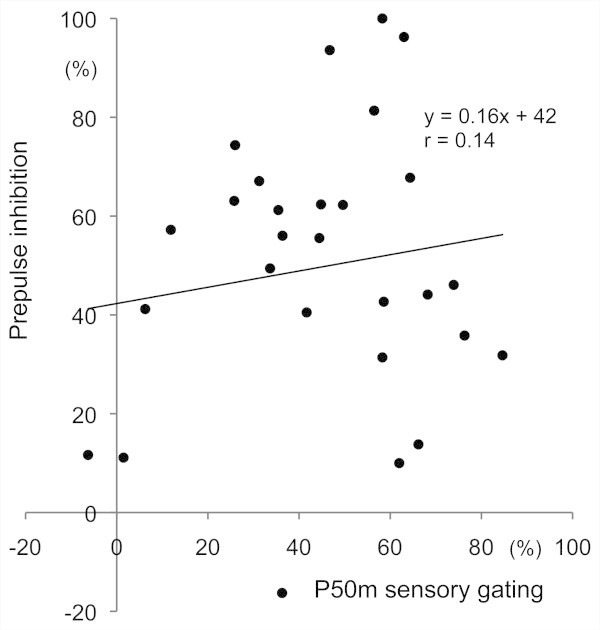
**Correlation between PPI and P50m sensory gating.** Data for the 60-ms Prepulse PPI are shown.

No significant difference was observed in the dipole location and orientation between Change-P50m and P50m (Figure 3C). The mean Talairach coordinates were -50, -23, 8 and 49, -19, 5 for P50m and -52, -21, 5 and 49, -15, 4 for Change-P50m. The amplitude of P50m for Click 1 and the amplitude of Change-P50m for the Test alone response were positively correlated (r = 0.61, p < 0.001; Figure 4). Concerning inhibition, P50m%inhibition was not significantly correlated with%PPI of the 30-ms Prepulse (r = 0.13), 60-ms Prepulse (0.14), 90-ms Prepulse (-0.06), or their average (0.09). The relationship between P50m sensory gating and PPI for the 60-ms Prepulse is shown in Figure 5.

## Discussion

Our previous study demonstrated that auditory change-related cortical activity peaking approximately 120 ms after the onset of an abrupt sound feature change (Change-N1m) was attenuated by a preceding weaker and briefer change stimulus (prepulse) in a similar manner to the PPI of startle responses (Inui et al. 2012). The present study confirmed that an earlier change-related component, Change-P50m, was also markedly inhibited by a prepulse. Similar to the PPI of the startle response in humans and rats (Swerdlow et al. 2004), the prepulse inhibited Change-P50m without eliciting clear cortical responses, which supports the presence of an inhibitory process. Although it is necessary to determine whether these phenomena and PPI of the startle reflex are regulated similarly, prepulse inhibition in the brain may be beneficial for studying the central mechanisms of the inhibitory process.

In the present study, we compared two measures using a similar index, the auditory evoked cortical response. The dipole location and orientation did not differ between P50m and Change-P50m, and the amplitude of Click 1 P50m and Test alone Change-P50m were also significantly correlated with each other, which suggested that they arise from a similar or same group of neurons. Regarding the similarity between P50m and Change-P50m, previous studies demonstrated auditory N1 in response to the sound onset (Onset-N1) to be a kind of Change-related cortical response elicited by the abrupt onset of a sound against silence (Nishihara et al. 2011), and that a positive correlation existed between the amplitude of Onset-N1m and Change-N1m evoked by a sound frequency change (r = 0.82) (Yamashiro et al. 2011). Although less is known about Change-P50m, our previous study showed the presence of a change-related endogenous component at 50 ~ 60 ms (Nakagawa et al. 2013). The present results support the hypothesis that onset P50m and Change-P50m share, at least in part, generating mechanisms and physiological significance. Therefore, it appeared likely that we could find a significant correlation between the two measures if the inhibitory process is similar. However, the results of the present study showed no clear relationship between P50m sensory gating and PPI, which confirmed previous studies using PPI of the startle response in healthy subjects (Schwarzkopf et al. 1993; Oranje et al. 1999; Light and Braff 2001; Brenner et al. 2004), patients with schizophrenia (Braff et al. 2007; Hong et al. 2007), and rats (Ellenbroek et al. 1999).

Regarding P50m sensory gating, the amplitude of the response to the first click depends on the sensitiveness of the subject to the abrupt sound change and the strength of sensory memory for the click of the preceding trial (Inui et al. 2010b). That is, stronger sensory memory attenuates the next response more strongly. However, this effect of sensory memory is negligible because the trial-trial interval of 10 s has been shown to be longer than the lifetime of sensory memory (Inui et al. 2010a). The amplitude for the second click depends on the strength of sensory memory for the first click, the subject’s own sensitiveness, and inhibitory action due to the first click if present. That is, more slowly fading sensory memory for the first click results in a smaller response. Taken together, the ratio of the two responses (S2/S1) largely depends on how much the first response contains change-related endogenous activity (sensitiveness) and the time course of sensory memory for the first click. For example, a subject with less change-related activity would show lower inhibition regardless of the status of sensory memory. A subject with quickly fading sensory memory would also show weak inhibition.

Regarding PPI of Change-P50m, the amplitude of the Test alone response depends on the sensitiveness of the subject’s change-detecting system similar to the Click 1 response for P50m sensory gating. The amplitude of the Prepulse + Test response depends on the strength of the inhibitory action elicited by the prepulse, the steepness of the rise in inhibitory action, and its lifetime. Some subjects exhibited stronger inhibition for the 30-ms, 60-ms, and 90-ms Prepulse in this order, which suggested that the 30-ms Prepulse exerted adequate inhibitory effects on the test response and these inhibitory effects became weaker with time in these subjects, as shown in a recent study on Change-N1m PPI (Inui et al. 2012). Others subjects exhibited the strongest inhibition with the 60-ms or 90-ms Prepulse, implying that 30 ms was too short to reach the maximum inhibition in these subjects. Taken together, the degree of the inhibition depends on the strength of the inhibitory action elicited by the Prepulse. The steepness of the rise in inhibitory action and its lifetime also affects the degree of the inhibition for each interval condition. Since our preliminary study showed that a prepulse with a prepulse-test interval longer than 200 ms only exerts a very weak effect on the test response, the lifetime of the inhibitory action in the present study appears to be far shorter than 500 ms. Therefore, an inhibitory process involved in P50m sensory gating appears weak if present.

To summarize, only the sensitiveness of the subject’s change-detecting system is a common factor between P50m sensory gating and PPI to determine the test response. The two measures appear to share few mechanisms for inhibition. Although the relationship between PPI of the startle response and PPI of the Change-related cortical response has not yet been clarified, previous studies on P50 sensory gating and PPI of the startle response supported the general hypothesis that the two measures reflect different inhibitory mechanisms. In clinical studies, it has been established that patients with schizophrenia show deficits in both P50 sensory gating and PPI of the startle response. This may be explained by these patients having reduced sensitivity to sensory changes, which is expected to result in a reduced test response for both measures and, therefore, in reduced inhibition. Our study showed that patients with schizophrenia had significantly weaker change-related auditory responses than those of healthy subjects (Ohoyama et al., unpublished data). Clinical studies showing that P50 gating and PPI were differently affected in a certain group of patients (Holstein et al. 2013) support the two measures being regulated differently. Pharmacological studies have also supported this by showing the different effects of a pharmacological substrate on the two measures (Mann et al. 2008).

One possible factor to influence the correlation between two measures is test-retest reliability of PPI and P50m gating. Although a recent study showed a good reliability of Change-N1m PPI (r^2^ = 0.45 ~ 0.49) (Kodaira et al. 2013), that of Change-P50m is not known. Since previous studies demonstrated that there are some techniques to improve the test-retest reliability of P50 sensory gating (Lu et al. 2007; Rentzsch et al. 2008), the reliability of Change-P50m PPI should be tested in future studies. If necessary, we should try to improve the reliability of Change-P50m PPI in order to use it as a biological marker of the sensory gating process.

Similar to previous studies on Change-N1m (Inui et al. 2010b, 2012), Change-P50m was larger in amplitude in the right hemisphere, implying right hemisphere dominance for change detection. In addition, the present study found that PPI was greater for the right hemisphere. In studies using startle blink reflexes, PPI was stronger for the right eye response in healthy subjects, however, this right side dominance disappeared in schizophrenics (Cadenhead et al. 2000). Although the precise relationship between hemispheric differences and startle response laterality remains unclear, it is interesting that both measures showed laterality. Since patients with schizophrenia and their relatives show less asymmetry of PPI (Cadenhead et al. 2000), future studies on hemispheric differences should progress our understanding of the biology and genetic background of this disease.

## Conclusion

Here, we demonstrated that 1) the auditory change-related P50m component was robustly inhibited by a prepulse, 2) a positive correlation existed between the baseline amplitudes of click-evoked P50m and Change-P50m and the neural origin did not differ between the two responses, which suggested that they were generated by a similar group of neurons in the auditory cortex, and 3) no correlation was found between P50m sensory gating and PPI of Change-P50m. Taken together, the present findings do not indicate that P50m sensory gating and PPI of Change-P50m reflect similar brain mechanisms. Because some diseases have been associated with an abnormality in both P50 gating and PPI, similarities and dissimilarities between these two measures still need to be elucidated in more detail. Future clinical studies on the PPI of Change-related cortical responses may provide us with important information on abnormalities in the inhibitory process in some diseases.

## Abbreviations

MEG: Magnetoencephalography
PPI: Prepulse inhibition
